# Circulating Sphingolipids and Glucose Homeostasis: An Update

**DOI:** 10.3390/ijms241612720

**Published:** 2023-08-12

**Authors:** Sarah Ali-Berrada, Jeanne Guitton, Sophie Tan-Chen, Anna Gyulkhandanyan, Eric Hajduch, Hervé Le Stunff

**Affiliations:** 1 Centre de Recherche des Cordeliers, INSERM, Sorbonne Université, F-75006 Paris, France; sarah.ali@inserm.fr (S.A.-B.); sophie.tan.crc@gmail.com (S.T.-C.); agg0708@yahoo.com (A.G.); 2 Institut Hospitalo-Universitaire ICAN, 75013 Paris, France; 3 Institut des Neurosciences Paris-Saclay, Université Paris-Saclay, CNRS UMR 9197, 91400 Saclay, France; jeanne.guitton@universite-paris-saclay.fr

**Keywords:** ceramides, sphingosine-1-phosphate, lipoproteins, extracellular vesicles, biomarkers

## Abstract

Sphingolipids are a family of lipid molecules produced through different pathways in mammals. Sphingolipids are structural components of membranes, but in response to obesity, they are implicated in the regulation of various cellular processes, including inflammation, apoptosis, cell proliferation, autophagy, and insulin resistance which favors dysregulation of glucose metabolism. Of all sphingolipids, two species, ceramides and sphingosine-1-phosphate (S1P), are also found abundantly secreted into the bloodstream and associated with lipoproteins or extracellular vesicles. Plasma concentrations of these sphingolipids can be altered upon metabolic disorders and could serve as predictive biomarkers of these diseases. Recent important advances suggest that circulating sphingolipids not only serve as biomarkers but could also serve as mediators in the dysregulation of glucose homeostasis. In this review, advances of molecular mechanisms involved in the regulation of ceramides and S1P association to lipoproteins or extracellular vesicles and how they could alter glucose metabolism are discussed.

## 1. Introduction

A sedentary lifestyle and overconsumption of cheap, calorie-dense foods has led to an unprecedented rise in obesity, representing a growing burden on public health. According to the World Health Organization (WHO), in 2016, more than 1.9 billion adults—people aged 18 and over—were overweight. Of this total, more than 650 million were obese [1,2]. Obesity is defined as excess body fat, leading to changes in adipose tissue that can impair health and reduce life expectancy [1,2]. When the storage capacity of the adipose tissue is impaired, ectopic storage of excess lipids is observed in peripheral tissues such as the liver and muscle and pancreatic β-cells [3,4,5,6]. In addition, low-grade inflammation in the adipose tissue will amplify adipocyte lipolysis, promoting increased plasma concentrations of fatty acids (FA), and thus, their ectopic deposition [4,7,8]. Obesity-related complications are numerous: respiratory failure, osteoarthritis, and some cancers are often associated with the development of cardiovascular disease and metabolic disorders such as non-alcoholic steatohepatitis (NASH) and type 2 diabetes (T2D) [9,10].

Ectopic lipid deposition in tissues contributes to the impairment of some cellular functions. In the liver, excessive fat accumulation promotes the development of non-alcoholic fatty liver disease (NAFLD), which explains why 70–90% of obese patients have NAFLD [10,11]. This pathology encompasses a broad spectrum of conditions ranging from the simple accumulation of fat in the liver, known as hepatic steatosis, to more advanced and inflammatory forms, such as the NASH, which can progress to hepatic fibrosis, cirrhosis, and eventually hepatocellular carcinoma [10,12]. Both obesity and NASH are epidemiologically linked to T2D, with the proportion of deaths due to diabetes having increased by 70% since 2000 [2]. T2D can lead to complications such as retinopathy, neuropathy, stroke, and myocardial infarction or arteritis of the lower limbs [9,13]. It is well known that people with both T2D and obesity develop insulin resistance in the liver, adipose tissue, and the skeletal muscle, characterized by reduced cellular response to insulin, leading to hyperglycemia [13]. This insulin resistance phenomenon results from an alteration in the insulin-responsive signaling pathway caused by ectopic lipid accumulation, thus demonstrating the crucial importance of insulin in the regulation of glucose homeostasis [4,14].

Tissue insulin resistance is multifactorial, and its origin is still the subject of much debate. At the muscle level in humans, there is an inverse correlation between increased intracellular FA concentrations and cell sensitivity to insulin [15]. However, the case of the athlete’s paradox contradicts this direct correlation. Athletes possess highly insulin-sensitive muscle tissue, but also high concentrations of intramuscular lipids stored in lipid droplets [16,17]. This suggests that it is not the intracellular accumulation of lipids per se that modulates insulin sensitivity, but rather the appearance and accumulation of some of their derivatives, such as sphingolipids [18].

Sphingolipids are a class of lipids characterized by an amino alcohol headgroup and fatty acid carbon chain. Sphingolipids play a structural role in membranes, and the specific balance between sphingolipid synthesis and degradation is essential for maintaining membrane cohesion. These biologically active molecules have been implicated in the regulation of various cellular processes, including inflammation, apoptosis, cell proliferation, autophagy, and lipid metabolism [4,19,20]. They have also emerged as key players in metabolic diseases such as diabetes, cardiovascular diseases, and neurodegenerative disorders [4,21,22].

Sphingolipids can act in different ways. They can be: (i) regulators of the properties of biophysical membranes, (ii) intracellular second messengers, or (iii) cofactors of the regulatory function of transmembrane proteins [23].

**Figure 1:**
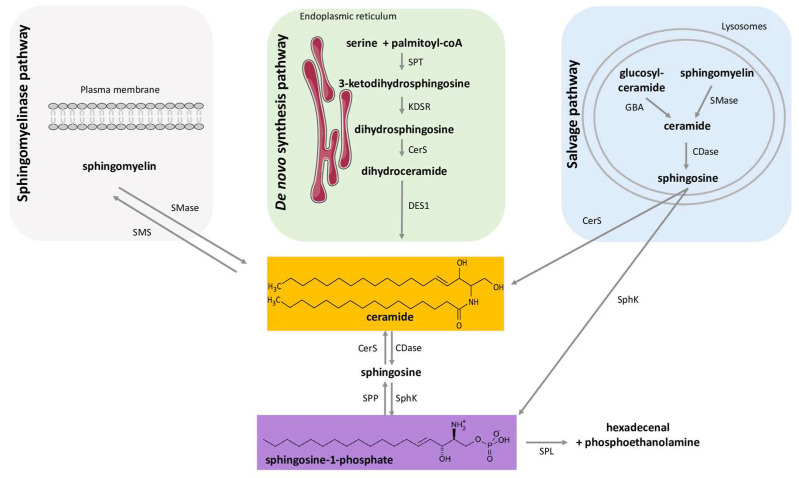
Regulation of ceramide and sphingosine-1-phosphate levels. Ceramide levels are regulated through three major metabolic pathways: the sphingomyelinase pathway, the de novo pathway, and the salvage pathway. S1P levels are regulated through the de-acylation of ceramide into sphingosine which is then phosphorylated. S1P can be recycled back to sphingosine or irreversibly degraded into hexadecenal and phosphoethanolamine. SPT: serine palmitoyl transferase; KDSR; 3-keto-dihydrosphingosine; CerS: ceramide synthases; DES1: dihydroceramide desaturase-1; GBA: acid β-glucosidase; SMase: sphingomyelinase; CDase: ceramidases; SMase: sphingomyelinase; SMS: sphingomyelin synthase; SphK: sphingosine kinase; SPP: lipid sphingosine phosphatase; SPL: sphingosine-1-phosphate lyase.

Ceramides act as backbones of sphingolipids such as sphingomyelin, sphingosine-1-phosphate (S1P), and gangliosides. Several metabolic pathways converge to produce ceramides: (i) the sphingomyelinase pathway that hydrolyses sphingomyelin to produce ceramides; (ii) the recycling pathway of complex sphingolipids in lysosomes; (iii) and the de novo synthesis pathway from saturated FA [7] (Figure 1). The de novo ceramide synthesis pathway located in the endoplasmic reticulum (ER) has been shown to be the major synthesis pathway activated under conditions of obesity and lipid excess [7]. Ceramides are synthesized de novo after condensation of L-serine with palmitate by serine palmitoyl transferase (SPT) (Figure 1). The formed 3-ketodihydrosphingosine is then rapidly metabolized into dihydrosphingosine by 3-ketodihydrosphingosine reductase. This is followed by acylation of dihydrosphingosine by ceramide synthase (CerS) to give dihydroceramide (dihydroCer). In mammals, six isoforms of CerS are expressed and are known for their ability to add an acyl-CoA chain of variable length (C14-C32) to dihydrosphingosine [24]. For example, CerS1 will produce C18-dihydroCer, and CerS5 and CerS6 mainly C16- dihydroCer, while CerS2 will produce long-chain dihydroCer such as C22- or C24- dihydroCer [24]. The different dihydroCer species are then desaturated by dihydroCer desaturase-1 (DES1) into ceramide species with different fatty acid chain lengths [24] (Figure 1). Several studies have shown that, depending on their fatty acid chain, different ceramide species can perform different functions in cells [24]. With the development of lipidomic methods, such as liquid chromatography coupled to mass spectrometry in tandem, to analyze the diversity of ceramide species, it has been well established that C16-ceramide and C18-ceramide species produced de novo in liver and muscle tissues play a predominantly deleterious role during the progression of T2D and NAFLD [25,26,27,28]. However, C22-, C24:1- and C24-ceramides would be considered more as ceramides exerting positive and protective actions in cells, especially in the liver [28,29,30,31]. Indeed, haploinsufficiency of CerS2, CerS isoform responsible for the synthesis of C22-, C24:1- and C24-ceramides, predisposed mice to diet-induced hepatic steatohepatitis and led to insulin resistance [28]. A number of recent reviews have dealt with the subject in depth [26,32,33].

Mechanisms of action of ceramides associated with insulin resistance are now quite well elucidated. In the short term, ceramides inhibit the insulin pathway via the activation of protein kinase Cζ (PKCζ) and/or protein phosphatase 2A (PP2A), which prevent activation of Akt in response to insulin [34,35,36]. In the longer term, ceramides indirectly inhibit insulin receptor substrate-1 (IRS1) via activation of two proteins: double-stranded RNA-dependent protein kinase (PKR) and c-Jun Kinase (JNK) [37].

In addition to ceramides, another sphingolipid species called S1P has also been shown to modulate glucose homeostasis and insulin response in cells/tissues [38]. In mammalian cells, ceramides act as precursors for S1P biosynthesis [39]. Indeed, ceramidases catalyze the transformation of ceramides into sphingosine, which then serves as a substrate for sphingosine kinases (SphK) to form S1P (Figure 1). Among the two forms of SphK (SphK1 and SphK2) [39], SphK1 expression is increased in insulin sensitive tissues but also in islets of Langerhans from mice under high fat diet (HFD) and in T2D patients [40], suggesting that this enzyme is involved in the onset of T2D associated with obesity. Conversely, S1P degradation is controlled either by two specific phosphate phosphohydrolases, which hydrolyze S1P to give sphingosine, or by the S1P-lyase (SPL), cleaving S1P into hexadecenal and phosphoethanolamine (Figure 1) [39]. However, and contrary to ceramides, the in vivo involvement of S1P in the development of insulin resistance and T2D remains controversial [41,42].

Although the involvement of de novo ceramide products in the inhibition of tissue insulin response and the development of metabolic diseases is now well demonstrated, increased-intracellular ceramide concentrations may not be exclusively due to local or intracellular de novo ceramide synthesis. Indeed, circulating ceramide concentrations are also strongly and rapidly amplified under lipotoxic conditions, as observed in obese diabetic patients compared with plasma ceramide concentrations in slim, healthy individuals [43,44]. Furthermore, plasma ceramides are associated with cardiovascular death in patients with stable coronary artery disease, acute coronary syndromes beyond low density lipoprotein (LDL)-cholesterol, and the onset of insulin resistance in non-diabetic individuals, 7 years before the development of the disease [44]. Another interesting fact is that circulating concentrations of dihydroceramides, a precursor of ceramides, are significantly increased in individuals who eventually develop diabetes, up to 9 years before the onset of the first symptoms of the disease [45]. A very recent study has shown that circulating concentrations of ceramides could also predict the durability of T2D remission after bariatric surgery [46,47]. This observation led the group of Summers to suggest that circulating ceramides, like cholesterol, could be used as biomarkers that predict cardiovascular events [48] and perhaps T2D. In the future, development of ceramide blood testing, which is only now performed by the Mayo Clinic, should help monitor patients [49].

In addition, Scherer and colleagues showed that both liver and adipose ceramide metabolism contributed to regulating plasma ceramide levels which are responsible for a dialogue between these tissues in order to modulate glucose metabolism and hepatic lipid uptake [50].

Ceramides are not the only sphingolipid derivatives to have been found in the circulation. S1P can also be secreted into the bloodstream [38]. Circulating S1P is mainly derived from erythrocytes and vascular endothelial cells in healthy subjects. Interestingly, plasma levels of S1P have been found to be higher in different models of insulin resistance in rodents, as well as in obese patients compared to non-obese individuals and correlates with the Homeostatic Model Assessment for Insulin Resistance (HOMA-IR), hemoglobin A1C (HbA1c), and body mass index [41].

Therefore, circulating sphingolipids could be an important lipid pool in the context of monitoring the development of diabetes, as some of them have been described as biomarkers to identify individuals at risk of developing insulin resistance [51], cardiovascular diseases [52], T2D [45,46], or associated hepatic steatosis [53]. This review aims to summarize the current literature on circulating sphingolipids, namely ceramides and S1P, involved in the regulation of glucose homeostasis in both human and animal models.

## 2. Ceramides and Lipoproteins

90% of circulating ceramides are associated with lipoproteins in the circulation [54]. The function of lipoproteins is to therefore allow the transport of hydrophobic lipids from the liver and intestine to other tissues [55]. Lipoproteins are spherical particles formed from proteins and specific lipids with a hydrophilic surface and a hydrophobic core [56]. The more protein-rich a lipoprotein is, the higher its density will be. Lipoproteins are thus divided into four classes, according to their density. Chylomicrons are the least dense lipoproteins. They are produced by the intestine and allow the transport of lipids brought by the food in the form of triglycerides (TG) and esterified cholesterol [57]. Very low density lipoproteins (VLDL) are released by the liver into the bloodstream and are rich in triglycerides (TG) [57]. Lipolysis of TG in VLDL leads to the generation of LDL which are rich in cholesterol and provide cholesterol to peripheral cells and tissues [56]. High-density lipoproteins (HDL) produced by the liver and the intestine play an important role in reversed cholesterol transport, the pathway by which cholesterol is removed from the tissue to the liver for elimination [57]. Sixty percent of ceramides are found in the LDL in healthy fasting human subjects [54]. Importantly, in T2D patients, LDL-transported ceramides concentrations are increased and negatively correlated with insulin sensitivity [54,58].

In the last decade, circulating ceramides have been associated with the development of atherosclerosis and cardiovascular diseases [59]. Moreover, plasma ceramides were able to predict cardiovascular death in patients with stable coronary artery disease and acute coronary syndromes beyond LDL-cholesterol [60]. This observation led the group of Summers to suggest that circulating ceramides, like cholesterol, could be used as biomarkers that predict cardiovascular events [48]. In addition, ceramide metabolism plays a central pro-atherogenic role in both the formation and maturation of atherosclerotic plaques through the regulation of oxidative stress [61]. Ceramide mediates insulin resistance through several mechanisms, including oxidative stress [62]. These observations could suggest that circulating ceramide observed in cardiometabolic patients could contribute to insulin resistance. More recent data suggests that ceramides could also act as non-autonomous signals to induce skeletal muscle ER stress [63]. This supports the idea that external sources of ceramides could contribute to insulin resistance. Indeed, studies have already shown that incubations of various insulin-responsive cells with exogenous short-chain ceramides (C2-ceramides) lead to insulin resistance [64,65,66,67,68,69,70,71], suggesting that an extracellular supply of ceramides may also be deleterious to the cell and may play an additive role to that of de novo produced ceramides. Thus, although increased intracellular ceramide concentrations have been associated with the onset and the development of metabolic diseases, this may not be exclusively due to intracellular de novo ceramide synthesis since several other studies have also demonstrated strong correlations between elevated circulating ceramides and insulin resistance and T2D [43,72,73,74,75,75,76,77,78].

**Figure 2:**
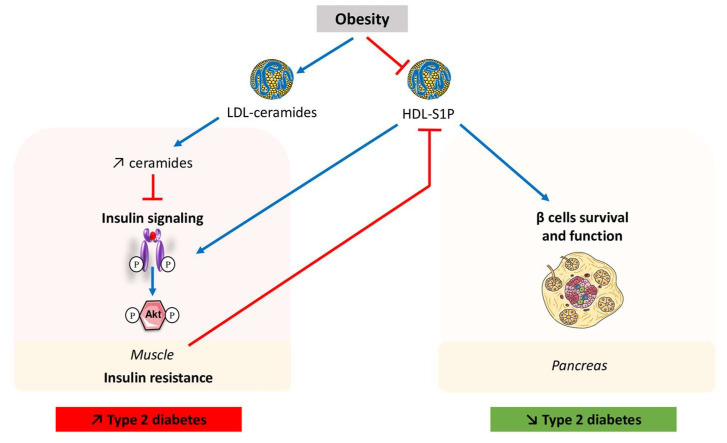
Role of sphingolipid transport by lipoproteins in the regulation of type 2 diabetes. Obesity and diabetes are associated with increased levels of ceramide-containing LDL, but also with decreased levels of HDL-S1P. LDL-ceramides were shown to inhibit insulin signaling (decreased phosphorylation of Akt) in muscle. HDL-S1P were shown to promote pancreatic cell function and survival. HDL-S1P can also enhance insulin signaling. Insulin resistance induced by obesity reduces HDL-S1P levels. Both LDL-ceramides and HDL-S1P display opposite actions on the development of T2D. HDL: high density lipoprotein; LDL: low density lipoprotein; S1P: sphingosine-1-phosphate. T2D: type 2 diabetes.

A very interesting study carried out a few years ago went further and also showed that circulating ceramide concentrations were increased in diabetic patients compared with normo-sensitive subjects [72]. At the same time, they also showed an increase in ceramide secretion by hepatocytes in diabetic patients compared to healthy subjects [72]. This result came as no surprise, since unlike in muscle and adipose tissue, ceramides do not accumulate in the liver [79]. Indeed, Watt and colleagues showed that lipid perfusion in healthy subjects resulted in rapid hepatic secretion of ceramides into the circulation, mainly within lipoproteins [31,79], thus protecting the liver from the deleterious effects of their intracellular accumulation [78]. To elucidate the physiological role of circulating LDL-ceramide in vivo, LDLs were enriched with C24-ceramide, the most abundant ceramide species in plasma and in LDL [54], and were infused intravenously into lean mice. LDL-C24-ceramide infusion reduced whole-body glucose clearance of the animals [72]. These data were confirmed in vitro, where myotubes treated with LDL-C24-ceramide displayed reduced insulin-stimulated glucose uptake and an inhibition of insulin signaling [72]. Thus, the deleterious involvement of LDL ceramides supports a functional role of circulating ceramide on insulin resistance (Figure 2).

Unlike LDL, there have been no studies suggesting a role for ceramides transported by VLDL or HDL in insulin resistance. As with LDL, C24-ceramide is the ceramide species found predominantly in both VLDL and HDL from healthy individuals [54]. One study showed that mice whose genes encoded for the VLDL receptor (VLDLR) were invalidated and who were fed a high fat diet (HFD) displayed better glucose tolerance and insulin sensitivity than wild-type mice fed the same diet [80], suggesting the possibility that VLDL-ceramides could play a role in modulating insulin sensitivity in these animals.

There are still very few studies showing the involvement of circulating ceramides associated with lipoproteins in insulin resistance and inflammation of peripheral tissues. These results are promising, but the deleterious involvement of C24-ceramides remains surprising as an increase of tissue C24-ceramide content is not shown to be correlated with insulin resistance [26] whereas, and as mentioned above, C16-/C18-ceramide species are [26]. Future experiments will be required to determine whether circulating ceramides and locally produced ceramides use similar signaling pathways to inhibit insulin signaling. In addition, further studies involving larger cohorts of patients will be needed to confirm the implication of these circulating sphingolipids in these processes.

## 3. Ceramides and Extracellular Vesicles

In addition to lipoproteins, ceramides can be secreted in the circulation under the form of extracellular vesicles (EVs). EVs are small, lipid bilayer-enclosed nanovesicles released by numerous cell types that play a critical role in intercellular communication. They are classified into three major types based on their biogenic, morphological, and biochemical properties: exosomes, micro-vesicles, and apoptotic bodies [81]. Exosomes, EVs of approximately 100 nm diameter, are generated within the endosomal system and fuse with the plasma membrane [82]. They can carry various biological materials, such as nucleic acids, proteins, and lipids that they can transfer to cell/tissue targets [82].

Since EVs secreted into the extracellular environment mainly transport cellular components from the cells in which they are synthesized, their characterization may have diagnostic and/or prognostic values for some metabolic diseases. Indeed, several studies have already demonstrated an increase in circulating EVs release from the liver under lipotoxic conditions [83,84,85].

**Figure 3:**
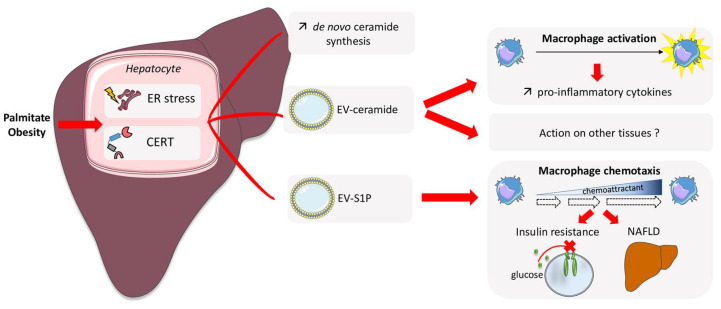
Role of sphingolipid transport by extracellular vesicles in the regulation of type 2 diabetes. Obesity (palmitate) was shown to stimulate the secretion of EVs enriched with ceramide by the liver. EV-ceramide production and secretion are mediated by increased ER stress and a CERT-dependent pathway. Secreted EV-ceramides favor the activation of macrophages, which contributes to NAFLD. Hepatocytes also secrete EV-enriched S1P, which stimulate macrophage chemotaxis, and therefore induce insulin resistance and NAFLD. Both EV-ceramide and EV-S1P secreted by the liver contribute to T2D development. CERT: ceramide transporter; ER: endoplasmic reticulum; EV: extracellular vesicle; NAFLD: non-alcoholic fatty liver disease; S1P; sphingosine-1-phosphate.

Palmitate, a saturated fatty acid, whose concentration was found to be very high in the liver and in the circulation in obesity-associated disorders, has been shown to increase the release of EVs from hepatocytes in an ER stress dependent manner, implicating the inositol requiring enzyme 1α (IRE1α)/X-box binding protein-1 pathway (Figure 3) [84,86,87]. Moreover, an IRE1α-regulated de novo ceramide synthesis was necessary for the palmitate-induced EV release in the circulation [86]. Likewise, the activation of IRE1α in the mouse liver stimulated the release of ceramide-enriched hepatocyte-derived EVs, and induced the accumulation of monocyte-derived macrophages (Figure 3) [88]. Furthermore, the EV content was increased in plasma from patients with NASH compared to control samples, and correlated with the histologic features of inflammation [88]. Inhibition of SPT in mice reduced EV release, supporting a ceramide involvement in the EV release process [88] (Figure 3). The ceramide transporter CERT has been shown to mediate the release of palmitate-stimulated ceramide enriched EVs from hepatocytes [89] (Figure 3). CERT was initially discovered as a lipid transfer protein that transports ceramides from the ER to other subcellular compartments such as the Golgi and mitochondria [90]. CERT was also shown to be present in multivesicular bodies such as EVs, and its knock-out in both hepatocytes and neuronal cells prevented ceramide transfer from the ER to EVs, as well as EV release from cells [89,91]. The importance of CERT in countering the toxic action of ceramides on the insulin response has also been demonstrated in muscle [92]. These data confirm the importance of this transporter in countering the accumulation of ceramides in tissues and thus preventing their deleterious action. Here in the liver, CERT is also thought to act against insulin resistance by rejecting de novo produced ceramides in EVs.

In addition to de novo synthesized ceramides, ceramides synthesized from sphingomyelin also regulate the biosynthesis and release of EVs, as inhibition of the enzymes involved in this pathway significantly reduces EV release [93]. Sphingomyelinases were shown to be involved in insulin resistance and β cell dysfunction and failure [94,95]. Whether the EV-ceramide produced by sphingomyelinases regulate insulin sensitivity is presently unknown.

As in hepatocytes, EVs can also be released by muscle cells upon lipotoxicity, and secreted exosomes can transfer lipids to adjacent cells [96]. More precisely, muscle-derived EVs are enriched in C16- and C22:1-ceramides [97]. In skeletal muscle, C16-ceramide is associated with insulin resistance [7] and impairs myogenic differentiation [98]. Thus, C16-ceramide released through EVs in response to lipotoxicity can participate in peripheral loss in insulin response.

EVs were also shown to be secreted by the adipose tissue [99]. A lipidomic analysis was recently performed on adipocyte-derived EVs derived from adipose tissue of lean and obese mice [100], and a specific ceramide enrichment was observed in adipocyte-derived EVs compared to the original adipose tissue (visceral adipose tissue), regardless of the metabolic status [100].

All these studies seem to suggest that EVs contain harmful materials from their producing cells. This could be a convenient way of preventing their cellular accumulation in deleterious conditions and protecting cells by modulating, for example, their immune response [101], and/or their repair capacity [102]. The organ protection through EV biogenesis and secretion was particularly highlighted in a study showing that the accumulation of adiponectin in EVs, a protein that plays an important role in cardiovascular protection [103], induced an amplification of EV biosynthesis and secretion and thus, a reduction in intracellular ceramide concentrations [104].

On the other hand, increased released of loaded EVs can be deleterious, as lipotoxic hepatocyte-derived EVs induced the expression and release of proinflammatory cytokines in macrophages [87], and thus, a decrease of the insulin response in hepatocytes [105]. In addition, it has been shown that hepatocyte-derived EVs play a key role in NAFLD pathogenesis [106]. However, few studies have demonstrated a direct deleterious action of the ceramides contained in EVs on glucose homeostasis and steatosis [86,88].

In addition, it has also been shown that EVs purified from the cerebrospinal fluid and frontal cortex of patients with Parkinson’s disease were heavily loaded with ceramides [107]. As ceramide reduction can rescue the neurodegenerative phenotype, it is possible that ceramide accumulation in EVs may contribute to nerve cell loss [108].

As with lipoproteins, involvement of ceramides contained in EVs in the progression of metabolic diseases is still open to debate. It is clear that ceramides play an important role in the biogenesis and secretion of EVs into the circulation. However, their involvement in the progression of T2D and its associated defects, such as insulin resistance and NASH, remains limited to hepatic inflammation, and further studies will therefore be needed to extend their role in the regulation of glucose homeostasis.

## 4. S1P and Lipoproteins

People suffering from insulin resistance who exhibit low HDL serum levels have been shown to be more susceptible to developing T2D. A correlation between HDL plasma concentrations and the abundance of small dense LDL is a higher risk factor for developing T2D [109]. HDL was shown to modulate the function and survival of pancreatic β cells. Rütti and colleagues demonstrated that HDL decreased both human and mouse islet cell apoptosis without influencing the function or the proliferation of these cells [110]. Furthermore, HDL protected islet cells from glucotoxicity and IL-1β-induced apoptosis [110]. These protective effects suggest a role in regulating β cell survival and hence, in the pathogenesis of T2D [110]. In agreement, Roehrich and colleagues demonstrated that the proapoptotic signaling of both LDL and VLDL was antagonized by HDL particles in islets isolated from mice [111]. The protective effects of HDL were mediated, in part, by the inhibition of caspase-3 cleavage and the activation of Akt [111]. In summation, balance between various lipoproteins are critical regulators of β cell survival and may therefore contribute to the β cell dysfunction observed during the development of T2D [111]. More recently, it has been shown that HDL could inhibit ER stress-induced apoptosis of pancreatic β-cells by activation of the hedgehog signaling pathway [112]. HDLs carry several types of lipids, but which ones are part of beneficial HDL effects on β cell survival and function is not completely understood.

There is some evidence suggesting that at least a part of HDL’s antidiabetic action involves apolipoprotein M (apoM) and its ligand S1P, two minor components of HDL [113]. S1P is a multifunctional lipid mediator and the agonist of five G protein-coupled receptors (GPCR), S1P 1 to 5 [114]. Serum apoM levels were lower in diabetic patients [115] and were negatively correlated with body composition indices such as body mass index, HbA1C, and HOMA-IR [116]. A study recently proposed that apoM/S1P association protected against the development of glucose intolerance and insulin resistance in HFD fed mice (Figure 2) [116]. In fact, apoM and S1P plasma levels were increased in mice with diet induced obesity. ApoM deficient mice exposed to a HFD had lowered plasma S1P levels and higher blood glucose levels, suggesting that apoM-knock-out (KO) mice were less tolerant of HFD-induced obesity [116]. In addition, insulin secretion during intraperitoneal glucose tolerance tests was attenuated in apoM-KO mice, indicating that apoM deficiency exacerbated insulin resistance during HFD (Figure 2). Conversely, HFD-fed mice overexpressing human apoM showed increased plasma levels of S1P, lower blood glucose levels, and less insulin resistance [116]. Likewise, adenovirus-mediated overexpression of apoM in C57BL6 mice displayed a better glucose tolerance through an enhanced insulin secretion mechanism [117]. Moreover, apoM-containing lipoproteins ameliorated insulin secretion from the pancreatic β cell line MIN6 [117]. The mechanism involved in the action of apoM could include a delay in the degradation of S1P, associated with increased levels of phosphorylated Akt, AMP-activated protein kinase (AMPK), and extracellular signal-regulated kinases (ERK) [117]. In line with these data, Goto-Kakizaki (GK) rats, a predisposed model of T2D, transfected with an adeno-associated virus (AAV) encoding for the rat apoM gene presented a higher apoM expression in pancreatic tissues [118]. ApoM overexpression in these rats decreased fasting blood glucose, improved glucose tolerance, and increased bodyweight and insulin levels [118]. Thus, apoM overexpression might improve both insulin secretion and insulin sensitivity in GK rats. Altogether, these studies demonstrated that the regulation of apoM/S1P levels is central for the development of glucose intolerance and insulin resistance in diabetes [116,117,118].

Mechanistically, phosphorylation of both Akt and AMPK, two well-known downstream targets of S1P1 and S1P3, was attenuated in the liver, adipose tissue, and skeletal muscle of apoM deficient mice during HFD. Conversely, their phosphorylation was enhanced in the liver and in the skeletal muscles of apoM overexpressed mice [117]. Treatment with S1P1 and S1P3 antagonists partially reversed the beneficial effects of apoM overexpression on fasting blood glucose levels and insulin resistance [117]. Consistent with these results, phosphorylation of Akt and AMPK, in the liver and in skeletal muscles was enhanced in apoM overexpressed mice, while treatment with VPC23019, an antagonist of S1P1/3, partially inhibited these protective effects [116]. The overexpression of apoM in GK rats induced higher Akt phosphorylation in the muscle, but not in the liver or adipose tissue [118]. These data highlighted that the complex apoM/S1P protects against the development of insulin resistance in the liver, adipose tissue, and skeletal muscle by activating Akt and AMPK signaling through S1P receptors 1 and 3 [116,117,118].

As stated above, low levels of HDL were observed in subjects with diabetes, explaining the higher prevalence of atherosclerotic diseases in these patients [109]. Insulin resistance itself led to a reduction in the production of apoM and a decrease in S1P/HDL levels (Figure 2) [119]. Both the quantity and quality of apoM were shown to be essential for binding S1P to HDL, and high-glucose conditions led to glycation of apoM, and thus to a reduction in apoM’s ability to bind S1P, resulting in its rapid degradation [120]. The maintenance of unglycosylated apoM is therefore essential for the apoM/S1P complex to exert its beneficial effects in tissues, suggesting that apoM may be a target of choice for the development of diabetic treatments.

Altogether, these studies provide evidence for a beneficial role of apoM/S1P complex in the regulation of glucose homeostasis. However, apoM/S1P complex could also induce obesity by negatively regulating brown adipose tissue activity [121]. Further research on its role in the development of T2D will be required.

## 5. S1P and Extracellular Vesicles

Circulating S1P is mainly associated with HDL or serum albumin [122]. Whether S1P could be found in EVs is under-explored. In the context of insulin resistance, Mahli and colleagues recently showed that palmitate-treated hepatocytes secreted EVs enriched not only with ceramide but also with S1P (Figure 3) [123]. EV-S1P were involved in macrophage chemotaxis through the S1P1 receptor, an important step in the development of liver inflammation induced by lipotoxicity (Figure 3) [123]. The presence of S1P in hepatic EVs was dependent on the activity of both SphK1 and SphK2 [123]. This opens up novel therapeutic strategies to limit insulin resistance. In the future, it would remain to determine whether other insulin sensitive tissues are able to secrete EV-S1P and what their roles in the regulation of glucose homeostasis are.

## 6. Conclusions and Open Questions

Under certain circumstances, such as lipotoxicity, intracellular concentrations of sphingolipids, which are produced de novo almost exclusively from saturated fatty acids, increase significantly. Numerous studies have shown that their accumulation in tissues such as skeletal muscle, liver, adipose tissue, and pancreatic β-cells often leads to pathologies. In organs such as the liver, some sphingolipid species, whose intracellular concentrations are increased, are secreted into the circulation in association with lipoproteins and/or in EVs. Although few studies to date show a direct action of the sphingolipids contained in these secreted products, it seems that both ceramides and S1P can modulate, positively or negatively, glucose metabolism in peripheral tissues [72,105,116,123].

Several studies have also shown important relationships between sphingolipid metabolites and the biogenesis/secretion/uptake of lipoproteins and/or EVs. What is interesting to note is that ceramides themselves, probably due to their site of synthesis (ER), appear to regulate the secretion of EVs that transport them [86,88,89]. It is therefore possible that an increase in their concentrations in lipoproteins in response to a lipotoxic environment could change the structural, physical, and functional properties of these macromolecules. Certain enzymes involved in sphingolipid biosynthesis pathways could also regulate EV secretion. Indeed, inhibition of neutral sphingomyelinase, an enzyme that converts sphingomyelins into ceramides, prevented the biosynthesis or release of some EV populations [124]. Further studies will therefore be needed to determine the specific roles of sphingolipid metabolic enzymes in the EV biogenesis/exocytosis. However, a difficulty in the elucidation of these roles is that sphingolipid metabolic enzymes are involved in different intracellular functions, so KO or over-expression approaches may simultaneously perturb EV biogenesis/and secretion, as well as other intracellular events.

Other proteins could also contribute to the accumulation of ceramide in lipoproteins. It has been found that the microsomal triglyceride transfer protein (MTP), the protein responsible for the assembly of lipoproteins, could transport sphingolipids, such as ceramide, into lipoproteins, and therefore determine circulating ceramide levels [125]. MTP deficiency had no effect on ceramide synthesis, but reduced the secretion from primary hepatocytes [125]. Since MTP expression is increased in diabetic subjects [126], it is therefore possible that MTP plays an important role in increasing circulating ceramide concentrations in this context. Another player could be the phospholipid transfer protein (PLTP) secreted by brown adipose tissue. A recent study showed that PLTP decreased circulating ceramide levels, a process associated with an improvement of glucose homeostasis [127].

Another interesting question would be to understand the role of sphingolipid metabolic enzymes and their products, ceramides, and S1P, in the regulation of EV uptake by targeted cells. Internalization occurs through either cell surface protein-protein or receptor-ligand interactions [128], and sphingolipid metabolites are known to regulate endocytosis pathway [129]. However, at present, it is not yet known whether the regulation of EV uptake by sphingolipid metabolites plays a role in the regulation of glucose homeostasis.

Finally, circulating concentrations of ceramides have been shown to predict the durability of T2D remission after bariatric surgery [46,47]. Furthermore, gut microbiota, key players in long-term glucose metabolism improvement after bariatric surgery [130], have been shown to regulate circulating ceramides [131]. One could therefore ask the question of the role of ceramides in this process. Do they only play a biomarker role in this prediction? Or do circulating ceramides have a deleterious functional role in the eventual rebound leading to reappearance of T2D? Future work will be very important to learn more about the possible functional role of sphingolipids present in lipoproteins and EVs in the regulation of metabolic diseases.

## Data Availability

Not applicable.
